# Complete Genome Sequence of a High Lipid-Producing Strain of *Mucor circinelloides* WJ11 and Comparative Genome Analysis with a Low Lipid-Producing Strain CBS 277.49

**DOI:** 10.1371/journal.pone.0137543

**Published:** 2015-09-09

**Authors:** Xin Tang, Lina Zhao, Haiqin Chen, Yong Q. Chen, Wei Chen, Yuanda Song, Colin Ratledge

**Affiliations:** 1 State Key Laboratory of Food Science and Technology, School of Food Science and Technology, Jiangnan University, Wuxi, P.R. China; 2 Synergistic Innovation Center for Food Safety and Nutrition, Wuxi, P.R. China; 3 Department of Biological Sciences, University of Hull, Hull, United Kingdom; Yonsei University, REPUBLIC OF KOREA

## Abstract

The genome of a high lipid-producing fungus *Mucor circinelloides* WJ11 (36% w/w lipid, cell dry weight, CDW) was sequenced and compared with that of the low lipid-producing strain, CBS 277.49 (15% w/w lipid, CDW), which had been sequenced by Joint Genome Institute. The WJ11 genome assembly size was 35.4 Mb with a G+C content of 39.7%. The general features of WJ11 and CBS 277.49 indicated that they have close similarity at the level of gene order and gene identity. Whole genome alignments with MAUVE revealed the presence of numerous blocks of homologous regions and MUMmer analysis showed that the genomes of these two strains were mostly co-linear. The central carbon and lipid metabolism pathways of these two strains were reconstructed and the numbers of genes encoding the enzymes related to lipid accumulation were compared. Many unique genes coding for proteins involved in cell growth, carbohydrate metabolism and lipid metabolism were identified for each strain. In conclusion, our study on the genome sequence of WJ11 and the comparative genomic analysis between WJ11 and CBS 277.49 elucidated the general features of the genome and the potential mechanism of high lipid accumulation in strain WJ11 at the genomic level. The different numbers of genes and unique genes involved in lipid accumulation may play a role in the high oleaginicity of strain WJ11.

## Introduction

Microbial oils have gathered interest as sources of nutritionally important polyunsaturated fatty acids (PUFAs) and as potential sources of biofuels [1,2]. γ-Linolenic acid (GLA; 18:3, n-6) is a critical PUFA and has proven beneficial for prevention and treatment of inflammatory disorders, diabetes, cardiovascular disorders, atopic dermatitis and cancers [3]. *Mucor circinelloides*, as a typical oleaginous filamentous fungus, has been widely used for lipid accumulation studies [4–6]. Moreover, it is rich in GLA and was the first microorganism to be used to commercially produce a single cell oil: a GLA-rich oil. The process began in 1985 but lasted only six years when high GLA-producing plant species came on to the market [7].

In oleaginous microorganisms, amounts of cellular lipid vary from 20% (w/w) up to more than 80% (w/w) cell dry weight [2,8]. *M*. *circinelloides* CBS 108.16 produces 20–25% (w/w) lipid [4,9] and strain CBS 277.49 accumulates no more than 15% (w/w) lipid [10,11]. A high lipid-producing strain *M*. *circinelloides* WJ11, however, has been isolated in our laboratory that produced up to 36% (w/w) lipid [11]. This is much higher than strains CBS 108.16 and CBS 277.49. In addition, the low lipid-producing strain, *M*. *circinelloides* CBS 277.49, had been sequenced by the Joint Genome Institute (JGI). Comparative genomic approaches now provide a powerful ability to identify multiple genes that are expressed differentially, especially between distinct microbial strains in same species [12].

Much work has been carried out to investigate the molecular mechanism of lipid accumulation in *M*. *cirinelloides* [4–6,9]; however, no studies have been done at the genomic level in *M*. *circinelloides*. Thus, in the present study, we sequenced the genome of high lipid-producing strain WJ11 and compared genome analyses between strains WJ11 and CBS 277.49 in order to understand their distinct lipid production patterns at genomic level. Together, the genome analysis presented in this study reveals new insights into the molecular basis for the lipid accumulation and provides rich genetic information that will be useful for the development of *M*. *circinelloides* as an oleaginous model fungus.

## Materials and Methods

### Strain and cultivation

*M*. *circinelloides* WJ11, previously isolated in our laboratory from soil at Jiangnan University [11], was used. 100 μl spore suspension (approx. 10^7^ spores/ml) of strain WJ11 was cultivated in 150 ml K & R medium [13] held in 1 L flasks equipped with baffles for 24 h at 30°C with shaking at 150 rpm and then used at 10% (v/v) to inoculate 2 L fermenters containing 1.5 L K & R media. Fermenters were controlled at 30°C with stirring at 700 rpm and aeration at 0.5 v/v min^-1^. The pH was maintained at 6.0 by auto-addition of 4 M KOH or 2 M H_2_SO_4_. *M*. *circinelloides* WJ11 was cultured for 16 h, and the mycelia were collected by filtration and then kept -80°C until DNA extraction.

### DNA sample preparation

Genomic DNA of *M*. *circinelloides* WJ11 was extracted using a modified protocol from Zhang et al. [14]. The mycelia of WJ11 was ground into powder in liquid N_2_ and then transferred into ice-cold, wash buffer [0.5 M sucrose, 80 mM KCl, 10 mM Trizma base, 10 mM EDTA, 1 mM spermidine, 1 mM spermine, 0.5% Triton X-100, 0.15% β-mercaptoethanol, pH 9.5]. The mixture was gently stirred for 10 min on ice, filtered, and then centrifuged at 1800 *g* for 20 min. The precipitate was resuspended in wash buffer, followed by centrifugation at 60 *g* for 2 min. The supernatant was collected and re-centrifuged at 1800 *g* for 20 min. The precipitate was washed three times and resuspended in homogenization buffer [0.5 M sucrose, 80 mM KCl, 10 mM Trizma base, 10 mM EDTA, 1 mM spermidine, 1 mM spermine, pH 9.5]. After centrifugation at 1800 *g* for 20 min, the precipitate was resuspended in extraction buffer [0.5 M NaCl, 10 M Trizma base, 20 mM EDTA, 1% SDS, 0.1% β-mercaptoethanol]. Proteinase K and RNase A were both added at 200 μg/ml and the mixture was held at 60°C for 1 h. The DNA sample was extracted twice with an equal volume of phenol/chloroform/isoamyl alcohol (25:24:1, by vol.), and then with chloroform/isoamyl alcohol (24:1 v/v). DNA was precipitated with 2.5 vol ice-cold ethanol and kept at -80°C overnight. After centrifuging at 12000 *g* for 30 min, the pellet was washed with 70% (v/v) ethanol, naturally dried and dissolved in TE buffer [10mM Tris/HCl, 1 mM EDTA, pH 8.0].

### Genome sequencing and assembly

Illumina (Sloxa) Genome Analyzer IIx and Roche (454) Genome Sequencer FLX systems were used for DNA sequencing of *M*. *circinellodies* WJ11. The 500 bp paired-end and 3 kb mate-pair DNA library were sequenced on the Illumina system. The 8 and 20 kb mate-pair DNA library was sequenced using the Roche system. Library preparation, sequencing and base calling were performed according to the manufacturer’s recommendations. Short reads generated from Illumina paired-end library were assembled by Velvet which adopts the de Bruijn graph data structure to construct contigs [15]. The contigs were then joined into scaffolds with Illumina mate-pair reads and Roche mate-pair reads. To get high confidence gene models of *M*. *circinellodies* WJ11, we employed an RNA-aided annotation strategy. Prediction of coding gene was accomplished with a modified PASA pipeline [16], AUGUSTUS [17], GlimmerHMM [18], GeneMark [19] and SNAP [20]. EVM (Evidence Modeler) [21] was used to merge the preliminary models. All predicted gene models were functionally annotated by their sequence similarity to genes and proteins in the NCBI nucleotide (Nt) and non-redundant UniProt/Swiss-Prot protein databases. The gene models were also annotated by their protein domains using InterProScan [22]. All genes were classified according to Gene Ontology (GO), eukaryotic orthologous groups (KOG) and KEGG metabolic pathways. The repeat sequences were masked throughout the genome using RepeatMasker (version 3.2.9) and the RepBase library (version 16.08).

### Comparative Genomics

Nucleotide and amino acid sequences of *M*. *circinelloides* CBS 277.49 were obtained from JGI (http://genome.jgi-psf.org/Mucci2/Mucci2.home.html). Genome comparisons were performed using the “progressive alignment” option available in the MAUVE program (version 2.3.1). Default scoring and parameters were used for generating the alignment. Synteny plots were generated by the MUMmer program using exact matching, clustering and alignment extension strategies based on the number of identical alignments between two genomes [23]. Pathway mapping was conducted by associating EC assignment and KO assignment with KEGG metabolic pathways based on BLAST search results. Unique gene acquisitions and losses between the two strains were clustered using the OrthoMCL method, and the similarity of proteins encoded by genes at a locus of interest (>50% identity at the predicted protein level) [24].

## Results and Discussion

### General features of *M*. *circirnelloides* WJ11 genome

**Fig 1:**
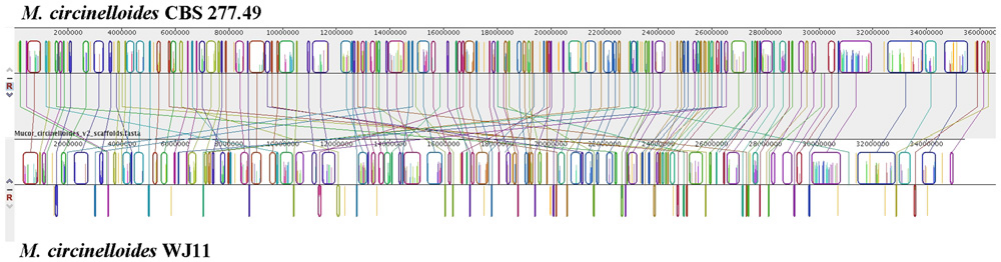
Alignment of the genomes from *M*. *circinelloides* WJ11 and CBS 277.49 using the MAUVE v. 2.3.1 software. Identically colored boxes, known as locally collinear blocks (LCBs), depict homologous regions in the two sequences. The vertical lines connecting the LCBs point to regions of homology between the two genomes. Numbers above the map indicate nucleotide positions.

**Fig 2:**
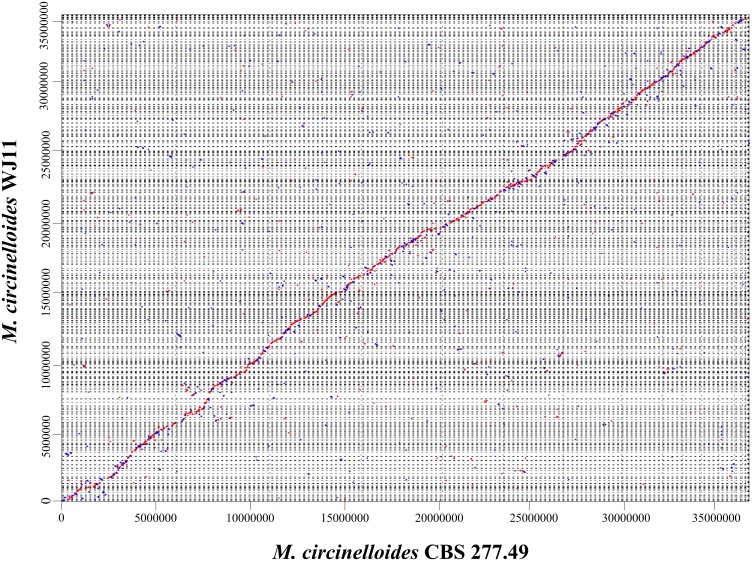
MUMmer-based genomic display between *M*. *circinelloides* WJ11 and CBS 277.49. Plus-strand matches are slanted from bottom left to upper right and are shown in red. Minus-strand matches are slanted from upper left to lower right and are shown in blue. The basepair window is at least 200 bp and the similarity ratio per dot is more than 50%. Numbers above the map indicate nucleotide positions.

Whole-genome sequencing of *M*. *circinelloides* WJ11 was performed with a combined strategy using Illumina and Roche sequencing technology and the sequence of *M*. *circinelloides* WJ11 was deposited in the public genome database (GenBank accession LGTF00000000). As shown in Table 1, the genome assembly size of stain WJ11 was 35.4 Mb, which did not exceed the value of *M*. *circinellodies* CBS 277.49 (36.6 Mb, sequenced by JGI). The G+C content of genome in strain WJ11 was 39.7%, which was lower than that of strain CBS 277.49 (42.2%). The numbers of predicted gene models and tRNAs in strain WJ11 were 10,973 and 177, respectively, which were both lower than strain CBS 277.49. However, strain WJ11 had longer average gene and protein length than strain CBS 277.49. Whole genome alignments with MAUVE revealed the presence of numerous blocks of homologous regions shared between the strains WJ11 and CBS 277.49 and MUMmer analysis showed genomes of these two strains were mostly co-linear (Figs 1 and 2).

**Table 1:** Characteristics of the gonomes of *M*. *circinelloides* WJ11 and CBS 277.49.

Genome feature	WJ11	CBS 277.49
**Size of assembled genome (Mb)**	35.4	36.6
**GC content (%)**	39.7%	42.2%
**Number of predicted gene models**	10973	11719
**tRNA genes**	177	237
**Average gene length (bp)**	1607.3	1429.3
**Average protein length (aa)**	458	379
**NR alignment**	8589	8691
**EC assignment**	2029	2066
**GO assignment**	5032	5036

### Reconstruction of central carbon and lipid metabolism pathways of *M*. *circinelloides*

**Fig 3:**
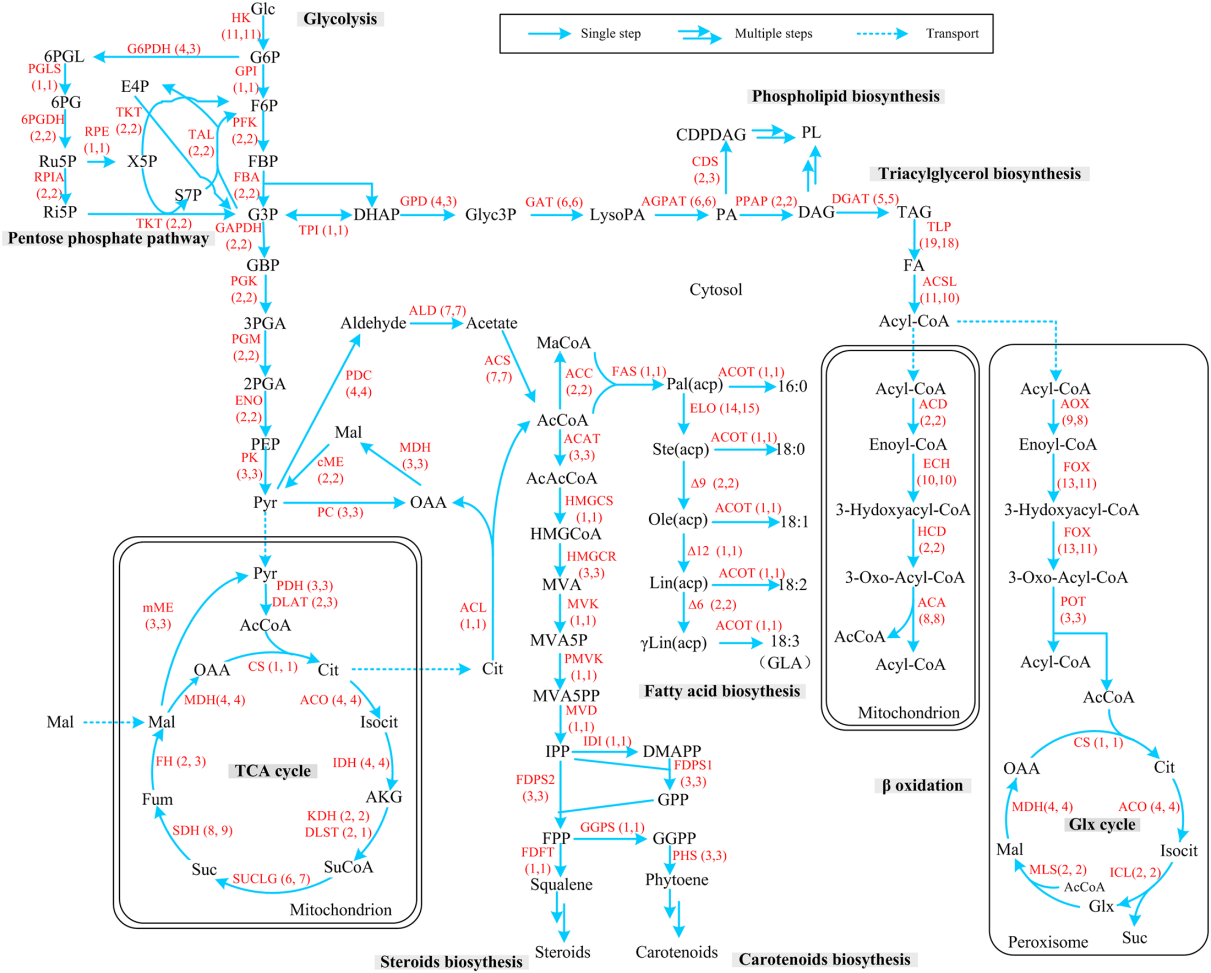
Reconstruction of central carbon and lipid metabolism pathways of *M*. *circinelloides* WJ11 and CBS 277.49. The metabolic network that was reconstructed from the KEGG pathway included glycolysis, pentose phosphate pathway, TCA cycle, fatty acid synthesis, sterodis and carotenoids biosynthesis, triacylglycerol and phospholipid biosynthesis, lipolysis β-oxidation and glyoxylate cycle. The number in parentheses indicated the number of genes encoding the enzymes in WJ11 and CBS 277.49, respectively. The abbreviations are included in S1 and S2 Tables.

Based upon the genome information, pathways of central carbon metabolism and lipid metabolism were reconstructed. These mapped the utilization of glucose, the biosynthesis of fatty acids, triacylglycerols, phospholipids, steroids and carotenoids, and β-oxidation (Fig 3). Furthermore, the numbers of genes that encode the enzymes in the pathways were compared between strains WJ11 and CBS 277.49.

The glycolysis pathway provides pyruvate, a key precursor for acetyl-CoA. The pentose phosphate pathway generates NADPH, which is a supplementary source to malic enzyme (ME) for providing reducing power for fatty acid biosynthesis [1]. The numbers of genes encoding the enzymes in glycolysis in WJ11 and CBS 277.49 were same. In the pentose phosphate pathway, most enzymes were encoded by the same number of genes in WJ11 and CBS 277.49. However, glucose-6-phosphate dehydrogenase (G6PDH) was encoded by four genes in WJ11 but was encoded by three genes in CBS 277.49. More genes encoding G6PDH possibly mean that there are greater opportunities for regulating the flux of carbon through the pentose phosphate pathway in WJ11 than in CBS 277.49 and this might indicate that WJ11 may have the potential to generate more NADPH than CBS 277.49 for fatty acid biosynthesis.

Indeed, our previous finding suggested that the pentose phosphate pathway was up-regulated which led to increased NADPH production in strain WJ11 compared with the low lipid-producing strain, CBS 277.49, during the lipid accumulation phase [11]. In addition, most enzymes in the glycolysis and pentose phosphate pathways were encoded by one to four genes, except for hexokinase (HK) which was encoded by up to 11 genes in both strains. Previous studies have indicated that *M*. *circielloides* has a higher glucose uptake rate than another fungus, *Mortierella alpina*, whose HK was encoded by only six genes [4,5,25]. The role of HK in the regulation of glucose uptake had been examined in *Aspergillus niger* and some other microorganisms [8,26]. Thus, our results suggest that active glucose uptake in *M*. *circinelloides* may be associated with its great number of HK-coding genes.

NADP^+^:ME plays a key role in supplying NADPH for fatty acid synthesis and desaturation [4,10,13,27–30]. In *M*. *circinelloides*, previous analysis has indicated the cytoplasmic NADP^+^:ME is encoded by two genes and mitochondrial NADP^+^:ME by one gene [31]. Our result showed the numbers and types of genes coding for NADP^+^:ME in WJ11 were the same as that of CBS 277.49. In addition, both WJ11 and CBS 277.49 had two genes encoding NAD^+^:ME.

The tricarboxylic acid (TCA) cycle is important in generating ATP, NAD(P)H and citrate. In the TCA cycle, some enzymes, such as succinyl-CoA ligase (SUCLG), succinate dehydrogenase (SDH) and fumarate hydratase (FH), were encoded by fewer genes in WJ11 than CBS 277.49. This indicated CBS 277.49 has an increased opportunity to regulate the TCA cycle and might therefore be linked with a more active TCA cycle in CBS 277.49 as shown by our previous work [11]. Although acetyl-CoA can also be produced from acetate when it is being used as a carbon source by cytoplasmic acetyl-CoA synthase (ACS), in oleaginous fungi and yeasts, acetyl-CoA is generated from the cleavage of citrate by ATP:citrate lyase (ACL) which is then the major provider of acetyl-CoA for lipid synthesis. The enzyme is possibly the rate-limiting reaction for fatty acid biosynthesis in some organisms [32–34]. In both strains WJ11 and CBS 277.49, ACL was encoded by single gene.

Acetyl-CoA carboxylase (ACC) converts acetyl-CoA to malonyl-CoA, and this conversion is a critical step in fatty acid biosynthesis. Fatty acid synthase (FAS) carries out enzymatic reactions necessary for the synthesis of saturated fatty acid (typically 16:0) from acetyl-CoA and malonyl-CoA. ACC was encoded by two genes and FAS was encoded by one gene in both WJ11 and CBS 277.49. Fatty acid delta 9 desaturase (Δ9) introduces the first double bond into a saturated fatty acid producing a monounsaturated fatty acid either palmitoleic acid [16:1(9)] or oleic acid [18:1(9)]. There are two genes encoding Δ9 desaturase in both strains. As *M*. *circinelloides* is a GLA-producing filamentous fungus, as expected, in both strains, there were two genes encoding fatty acid delta-12 desaturase (Δ12) [to convert oleic acid to linoleic acid, 18:2(9,12)] and one gene encoding fatty acid delta-6 desaturase (Δ6) to convert linoleic acid into γ-linolenic acid [18:3(6,9,12)]. *M*. *circinelloides* has also been used as a model organism for carotenoid biosynthesis [35,36]. Indeed, the enzymes (e.g., geranylgeranyl pyrophosphate synthases, phytoene synthase) related to carotenoids biosynthesis were identified in WJ11 and CBS 277.49, revealing the genetic basis for the formation of the yellow-colored pigments by this organism.

Most enzymes involved in the biosynthesis of steroids, phospholipid and triacylglycerol in both strains were encoded by equal number of genes. However, glycerol-3-phosphate dehydrogenase (GDP) was encoded by more genes, while phosphatidate cytidylyltransferase (CDS) was encoded by fewer genes in WJ11 than in CBS 277.49. These differences might affect the flux of glycerol into the biosynthesis of triacylglycerols. Many genes encoding enzymes involved in fatty acid β-oxidation in the peroxisomes and mitochondrion were identified in both strains which indicates that they will have an active β-oxidation system though little has been reported on this activity in filamentous fungi in general.

### Genome comparison and unique genes identification between strain WJ11 and CBS 277.49

OrthoMCL clustering procedure was used to find unique genes of each strain and these unique genes were subjected to BLAST in NCBI database. Strains WJ11 and CBS 277.49 contained, respectively, 152 and 186 unique genes other than hypothetical proteins (S3 and S4 Tables). These differences may reflect different functions in cell growth and cell metabolism in these two strains.

Strain WJ11 grows faster than CBS 277.49 during the initial stages [11]. Fungal cell walls consist of various glucans and chitin, and require chitinase to enlarge the cell wall surface area during hyphal growth [37]. In strain WJ11, some unique genes (e.g., chitin synthase, chitinase, chitin deacetylase) were directly involved in chitin metabolism, which may play a role in the differential cell growth of the strains. The unique gene in WJ11 encoding phosphatidylserine decarboxylase is essential for cell wall integrity and virulence [38] and that encoding protein-serine/threonine phosphatase is crucial for cell growth [39]. Thus, the distinct cell growth between WJ11 and CBS 277.49 may be related with the above unique genes. In addition, Ca^2+^-transporting ATPase, which is essential for Ca^2+^ homeostasis and related to salt tolerance [12,40], was encoded by an unique gene in CBS 277.49.

Carbohydrate metabolism is important for the fatty acid biosynthesis. Acetyl-CoA, as the precursor of fatty acids, and NADPH, as the reducing power, are critical for fatty acid biosynthesis [1,8]. Comparison of biochemical activities between the two strains have indicated that a greater flux of carbon through the TCA cycle rather than to acetyl-CoA for lipid biosynthesis in CBS 277.49 may explain its low oleaginicity [11]. Indeed, some unique genes encoding glycolytic enzymes (e.g., glyceraldehyde-3-phosphate dehydrogenase, pyruvate dehydrogenase, pyruvate carboxylase) were identified in WJ11. Furthermore, there are several unique genes encoding the enzymes involved in TCA cycle, such as succinate dehydrogenase, succinyl-CoA ligase and dihydrolipoyllysine acetyltransferase in CBS 277.49. These unique genes might therefore be associated with the greater flux of carbon to TCA cycle as demonstrated by our previous work [11]. Notably, glucose-6-phosphate dehydrogenase (G6PDH), is the enzyme providing NADPH in pentose phosphate pathway, was encoded by an unique gene in WJ11. Previous research has indicated that NADPH generated from pentose phosphate pathway plays a key role in lipid accumulation in maize embryos, *Chlorella protothecoides* and *Yarrowia lipolytica* [41–43]. Thus, this unique gene encoding G6PDH may play a role in providing additional NADPH for lipid accumulation in WJ11.

Many unique genes in WJ11 and CBS 277.49 are involved in lipid metabolism. Both WJ11 and CBS 277.49 have the unique genes encoding glycerol-3-phosphate dehydrogenase (GPD), glycerol-3-phosphate *O-*acyltransferase (GAT) and 1-acylglycerol-3-phosphate acyltransferase (AGPAT). These enzymes regulate the lipid biosynthesis in other organisms: for example, in some plants, over-expression of GPD increased lipid accumulation [44] and GAT is the first enzyme in the pathway for the de novo synthesis of membrane and storage lipids [45]. AGPAT is involved in final steps of triacylglycerol biosynthesis [46].

Triacylglycerol lipase (TLP) releases fatty acids from TAGs and cleaves fatty acyl groups at each of the *sn*1-, *sn*2-, and *sn*3- positions [47]. 3-Ketoacyl-CoA thiolase (ACA, peroxisomal; or POT, mitochondrial) catalyzes a key step in fatty acid β-oxidation [48]. Thus, the unique genes encoding TPL and ACA (or POT) might have an effect on the lipid catabolism in these strains. Long chain fatty acid:CoA ligase takes part in long fatty acid biosynthesis (e.g., palmitic acid) [49] and, indeed, a unique gene encoding this enzyme was identified in WJ11. Taken together, these unique genes involved in lipid metabolism in WJ11 and CBS 277.49 could make a contribution to their distinct patterns of lipid accumulation.

## Conclusion

The genome of *M*. *circinelloides* WJ11, which accumulates up to 36% lipid (w/w, CDW), was sequenced and compared with the low lipid-producing strain CBS 277.49 (15% lipid, w/w, CDW), which had been sequenced by the JGI. The general features of these two strains suggested that there is strong similarity at the level of gene order and gene identity. Whole genome alignments with MAUVE revealed the presence of numerous blocks of homologous regions, and MUMmer analysis showed genomes of these two strains were mostly co-linear. Furthermore, the central carbon and lipid metabolism pathways of these two strains were reconstructed and the numbers of genes encoding the enzyme involved in these pathways were compared. Many unique genes identified for each strain were involved in cell growth and metabolism. These unique genes may be associated with the differential growth and lipid accumulation in these two strains. Taken together, the genome sequence of WJ11 and the comparative genomes analysis between WJ11 and CBS 277.49 elucidated the general feature of genome and the difference at the genome level that is potentially related to lipid accumulation, and this lays the basis of future explorations of both basic and applied biological problems in lipid metabolism.

## Supporting Information

S1 Table Abbreviations of substrates in Fig 3. (DOCX)

S2 Table Abbreviations of the enzymes in Fig 3. (DOCX)

S3 Table Listing of *M*. *circinelloides* WJ11 specific genes as compared to *M*. *circinelloides* CBS 277.49. (DOCX)

S4 Table Listing of *M*. *circinelloides* CBS 277.49 specific genes as compared to *M*. *circinelloides* WJ11. (DOCX)
