# Supplementary material for: Complete Genome Sequence of a High Lipid-Producing Strain of Mucor circinelloides WJ11 and Comparative Genome Analysis with a Low Lipid-Producing Strain CBS 277.49
Source: PLoS One. 2015 Sep 9;10(9):e0137543. doi: 10.1371/journal.pone.0137543 (PMC4564205; doi:10.1371/journal.pone.0137543)
Supplement: S1 Table — (DOCX) [file pone.0137543.s001.docx]

**S1 Table. Abbreviations of substrates in Fig. 3.**

| **Abbreviations** | **Substrates** |
| --- | --- |
| Glc | glucose |
| G6P | glucose 6-phosphate |
| F6P | Fructose 6-phosphate |
| FBP | fructose 1,6-bisphosphate |
| G3P | glyceraldehyde 3-phosphate |
| 6PGL | 6-phosphogluconolactone |
| 6PG | 6-phosphogluconate |
| Ru5P | ribulose 5-phosphate |
| X5P | xylulose 5-phosphate |
| Ri5P | ribose 5-phosphate |
| E4P | erythrose 4-phosphate |
| S7P | sedoheptulose 7-phosphate |
| GBP | glycerate 1,3-bisphosphate |
| 3PGA | 3-phosphoglyceric acid |
| 2PGA | 2-phosphoglyceric acid |
| PEP | phosphoenolpyruvate |
| Pyr | pyruvate |
| AcCoA | acetyl coenzyme A |
| Cit | citrate |
| Isocit | isocitrate |
| AKG | 2-ketoglutarate |
| SuCoA | succinyl coenzyme A |
| Suc | succinate |
| Fum | fumarate |
| Mal | malate |
| OAA | oxaloacetate |
| MaCoA | malonyl coenzyme A |
| Pal(acp) | palmitoyl-(acyl carrierprotein protein) |
| Ste(acp) | stearidonoyl-(acyl carrierprotein protein) |
| Ole(acp) | oleoyl-(acyl carrierprotein protein) |
| Lin(acp) | linolenoyl-(acyl carrierprotein protein) |
| γLin(acp) | γ-linolenoyl-(acyl carrierprotein protein) |
| GLA | γ-linolenic acid |
| AcAcCoA | acetoacetyl coenzyme A |
| HMGCoA | 3-hydroxy-3-methylglutaryl coenzyme A |
| MVA | mevalonate |
| MVA5P | mevalonate-5-phosphate |
| MVA5PP | mevalonate-5-pyrophosphate |
| IPP | 3-isopentenyl pyrophosphate |
| DMAPP | dimethylallyl diphosphate |
| GPP | geranyl pyrophosphate |
| FPP | farnesyl pyrophosphate |
| GGPP | geranylgeranyl pyrophosphate |
| DHAP | dihydroxyacetonephosphate |
| Glyc3P | glycerol 3-phosphate |
| LysoPA | lysophosphatidic acid |
| PA | phosphatidic acid |
| DAG | diacylglycerol |
| TAG | triacylglycerol |
| CDPDAG | cytidine diphosphate-diacylglycerol |
| PL | phospholipid |
| FA | fatty acid |
| Glx | glyoxylate |
