# Supplementary material for: Complete Genome Sequence of a High Lipid-Producing Strain of Mucor circinelloides WJ11 and Comparative Genome Analysis with a Low Lipid-Producing Strain CBS 277.49
Source: PLoS One. 2015 Sep 9;10(9):e0137543. doi: 10.1371/journal.pone.0137543 (PMC4564205; doi:10.1371/journal.pone.0137543)
Supplement: S2 Table — (DOCX) [file pone.0137543.s002.docx]

**S2 Table. Abbreviations of the enzymes in Fig. 3.**

| **Abbreviations** | **EC number** | **enzymes** |
| --- | --- | --- |
| HX | 2.7.1.1 | hexokinase |
| GPI | 5.3.1.9 | glucose-6-phosphate isomerase |
| PFK | 2.7.1.11 | phosphofructokinase |
| FBA | 4.1.2.13 | fructose-bisphosphate aldolase |
| TPI | 5.3.1.1 | triose-phosphate isomerase |
| GAPDH | 1.2.1.12 | glyceraldehyde 3-phosphate dehydrogenase |
| PGK | 2.7.2.3 | phosphoglycerate kinase |
| PGM | 5.4.2.1 | phosphoglycerate mutase |
| ENO | 4.2.1.11 | enolase |
| PK | 2.7.1.40 | pyruvate kinase |
| PDH | 1.2.4.1 | pyruvate dehydrogenase |
| DLAT | 2.3.1.12 | dihydrolipoamide acetyltransferase |
| CS | 2.3.3.1 | citrate synthase |
| ACO | 4.2.1.3 | aconitate |
| IDH | 1.1.1.41 | isocitrate dehydrogenase |
| KDH | 1.2.4.2 | 2-ketoglutarate dehydrogenase |
| DLST | 2.3.1.61 | dihydrolipoamide succinyltransferase |
| SUCLG | 6.2.1.4/ 6.2.1.5 | succinyl-CoA ligase |
| SDH | 1.3.5.1 | succinate dehydrogenase |
| FH | 4.2.1.2 | fumarate hydratase |
| MDH | 1.1.1.37 | malate dehydrogenase |
| ACL | 2.3.3.8 | ATP-citrate lyase |
| cME | 1.1.1.40 | malic enzyme, cytoplasmic |
| mME | 1.1.1.39/1.1.1.40 | malic enzyme, mitochondrial |
| PC | 6.4.1.1 | pyruvate carboxylase |
| PDC | 4.1.1.1 | pyruvate decarboxylase |
| ALD | 1.2.1.3 | aldehyde dehydrogenase |
| ACS | 6.2.1.1 | acetyl-CoA synthetase |
| ACC | 6.4.1.2 | acetyl-CoA carboxylase |
| FAS | 2.3.1.86 | fatty acid synthase |
| ACOT | 3.1.2.27 | acyl-CoA thioesterase |
| ELO | 2.3.3.- | fatty acid elongase |
| △9 | 1.14.19.1 | fatty acid delta 9 desaturase |
| △12 | 1.14.19.6 | fatty acid delta 12 desaturase |
| △6 | 1.14.19.3 | fatty acid delta 6 desaturase |
| ACAT | 2.3.1.9 | acetyl-CoA C-acetyltransferase |
| HMGCS | 2.3.3.10 | hydroxymethylglutaryl-CoA synthase |
| HMGCR | 1.1.1.34 | hydroxymethylglutaryl-CoA reductase |
| MVK | 2.7.1.36 | mevalonate kinase |
| PMVK | 2.7.4.2 | phosphomevalonate kinase |
| MVD | 4.1.1.33 | diphosphomevalonate decarboxylase |
| IDI | 5.3.3.2 | isopentenyl-diphosphate delta-isomerase |
| PDPS1 | 2.5.1.1 | farnesyl diphosphate synthase 1 |
| PDPS2 | 2.5.1.10 | farnesyl diphosphate synthase 2 |
| FDFT | 2.5.1.21 | farnesyl-diphosphate farnesyltransferase |
| GGPS | 2.5.1.29 | geranylgeranyl pyrophosphate synthases |
| PHS | 2.5.1.32 | phytoene synthase |
| ICL | 4.1.3.1 | isocitrate lyase |
| MLS | 2.3.3.9 | malate synthase |
| G6PDH | 1.1.1.49 | glucose-6-phosphate dehydrogenase |
| PGLS | 3.1.1.31 | 6-phosphogluconolactonase |
| 6PGDH | 1.1.1.44 | 6-phosphogluconate dehydrogenase |
| RPE | 5.1.3.1 | ribulose-phosphate 3-epimerase |
| RKIA | 5.3.1.6 | ribose 5-phosphate isomerase A |
| TAL | 2.2.1.2 | transaldolase |
| TKT | 2.2.1.1 | transketolase |
| GPD | 1.1.1.8 | glycerol-3-phosphate dehydrogenase |
| GAT | 2.3.1.15 | glycerol-3-phosphate O-acyltransferase |
| AGPAT | 2.3.1.51 | 1-acylglycerol-3-phosphate acyltransferase |
| PPAP | 3.1.3.4 | phosphatic acid phosphatase |
| CDS | 2.7.7.41 | phosphatidate cytidylyltransferase |
| DGAT | 2.3.1.20 | diacylglycerol O-acyltransferase |
| TLP | 3.1.1.3 | triacylglycerol lipase |
| ACSL | 6.2.1.3 | acyl-CoA synthetase |
| AOX | 1.3.3.6 | acyl-CoA oxidase |
| ACD | 1.3.99.- | acyl-CoA dehydrogenase |
| FOX | 4.2.1.- /1.1.1.- | multifunctional beta-oxidation protein |
| ECH | 4.2.1.17 | enoyl-CoA hydratase |
| HCD | 1.1.1.35 | 3-hydroxyacyl-CoA dehyrogenase |
| POT | 2.3.1.9 | 3-ketoacyl-coA thiolase A, peroxisomal |
| ACA | 2.3.1.16 | 3-ketoacyl-CoA thiolase, mitochondrial |
