# Supplementary material for: Complete Genome Sequence of a High Lipid-Producing Strain of Mucor circinelloides WJ11 and Comparative Genome Analysis with a Low Lipid-Producing Strain CBS 277.49
Source: PLoS One. 2015 Sep 9;10(9):e0137543. doi: 10.1371/journal.pone.0137543 (PMC4564205; doi:10.1371/journal.pone.0137543)
Supplement: S3 Table — (DOCX) [file pone.0137543.s003.docx]

**S3 Table. Listing of *M. circinelloides* WJ11 specific genes as compared to *M. circinelloides* CBS 277.49.**

| **Gene ID** | **EC number** | **Enzyme** |
| --- | --- | --- |
| **Cell growth related genes** |  |  |
| evm.model.scaffold00015.54 | 2.4.1.16 | chitin synthase |
| evm.model.scaffold00018.52 | 2.4.1.16 | chitin synthase |
| evm.model.scaffold00100.27 | 2.4.1.16 | chitin synthase |
| evm.model.scaffold00175.21 | 2.4.1.16 | chitin synthase |
| evm.model.scaffold00200.9 | 2.4.1.16 | chitin synthase |
| evm.model.scaffold00203.13 | 2.4.1.16 | chitin synthase |
| evm.model.scaffold00230.15 | 2.4.1.16 | chitin synthase |
| evm.model.scaffold00094.6 | 3.1.3.16 | protein-serine/threonine phosphatase |
| evm.model.scaffold00004.80 | 3.2.1.14 | chitinase |
| evm.model.scaffold00100.9 | 3.2.1.14 | chitinase |
| evm.model.scaffold00045.49 | 3.5.1.41 | chitin deacetylase |
| evm.model.scaffold00006.57 | 4.1.1.65 | phosphatidylserine decarboxylase |
| evm.model.scaffold00030.45 | 4.1.1.65 | phosphatidylserine decarboxylase |
| **Carbohydrate metabolism** |  |  |
| evm.model.scaffold00296.11 | 1.1.1.14 | L-iditol 2-dehydrogenase |
| evm.model.scaffold00402.1 | 1.1.1.49 | glucose-6-phosphate dehydrogenase |
| evm.model.scaffold00230.13 | 1.2.1.12 | glyceraldehyde-3-phosphate dehydrogenase |
| evm.model.scaffold00105.14 | 1.2.4.1 | pyruvate dehydrogenase |
| evm.model.scaffold00016.49 | 2.3.1.61 | dihydrolipoamide succinyltransferase |
| evm.model.scaffold00752.1 | 2.3.1.61 | dihydrolipoamide succinyltransferase |
| evm.model.scaffold00129.11 | 2.7.1.69 | protein-Npi-phosphohistidine-sugar phosphotransferase |
| evm.model.scaffold00149.10 | 2.7.1.105 | 6-phosphofructo-2-kinase |
| evm.model.scaffold00003.54 | 2.7.7.13 | mannose-1-phosphate guanylyltransferase |
| evm.model.scaffold00748.1 | 2.7.7.13 | mannose-1-phosphate guanylyltransferase |
| evm.model.scaffold00101.12 | 3.2.1.20 | alpha-glucosidase |
| evm.model.scaffold00317.7 | 3.2.1.20 | alpha-glucosidase |
| evm.model.scaffold00635.1 | 3.2.1.20 | alpha-glucosidase |
| evm.model.scaffold00165.1 | 3.5.1.25 | N-acetylglucosamine-6-phosphate deacetylase |
| evm.model.scaffold00434.2 | 5.3.1.8 | mannose-6-phosphate isomerase |
| evm.model.scaffold00058.14 | 6.4.1.1 | pyruvate carboxylase |
| **Lipid metabolism** |  |  |
| evm.model.scaffold00409.1 | 1.1.1.8 | glycerol-3-phosphate dehydrogenase (NAD^+^) |
| evm.model.scaffold00353.5 | 1.1.5.3 | glycerol-3-phosphate dehydrogenase |
| evm.model.scaffold00208.8 | 1.3.1.70 | Delta14-sterol reductase |
| evm.model.scaffold00192.13 | 1.3.1.71 | Delta24(241)-sterol reductase |
| evm.model.scaffold00192.14 | 1.3.1.71 | Delta24(241)-sterol reductase |
| evm.model.scaffold00384.1 | 2.3.1.15 | glycerol-3-phosphate 1-O-acyltransferase |
| evm.model.scaffold00057.11 | 2.3.1.22 | 2-acylglycerol O-acyltransferase |
| evm.model.scaffold00127.7 | 2.3.1.26 | sterol O-acyltransferase |
| evm.model.scaffold00570.1 | 2.3.1.51 | 1-acylglycerol-3-phosphate O-acyltransferase |
| evm.model.scaffold00119.26 | 2.3.1.9 | 3-ketoacyl-CoA thiolase |
| evm.model.scaffold00101.21 | 2.7.8.1 | ethanolaminephosphotransferase |
| evm.model.scaffold00041.20 | 3.1.1.3 | triacylglycerol lipase |
| evm.model.scaffold00009.32 | 3.1.1.3 | triacylglycerol lipase |
| evm.model.scaffold00110.32 | 3.1.1.3 | triacylglycerol lipase |
| evm.model.scaffold00247.1 | 3.1.4.4 | phospholipase D |
| evm.model.scaffold00032.4 | 6.2.1.3 | long-chain-fatty-acid: CoA ligase |
| evm.model.scaffold00563.1 | 6.2.1.3 | long-chain-fatty-acid: CoA ligase |
| **Others** |  |  |
| evm.model.scaffold00239.16 | 1.1.1.169 | 2-dehydropantoate 2-reductase |
| evm.model.scaffold00042.2 | 1.6.2.2 | cytochrome-b5 reductase |
| evm.model.scaffold00016.69 | 1.9.3.1 | cytochrome-c oxidase |
| evm.model.scaffold00238.2 | 1.9.3.1 | cytochrome-c oxidase |
| evm.model.scaffold00238.3 | 1.9.3.1 | cytochrome-c oxidase |
| evm.model.scaffold00377.1 | 1.10.2.2 | quinol-cytochrome-c reductase |
| evm.model.scaffold00811.1 | 1.10.2.2 | quinol-cytochrome-c reductase |
| evm.model.scaffold00286.2 | 1.10.3.3 | L-ascorbate oxidase |
| evm.model.scaffold00038.21 | 1.11.1.15 | peroxiredoxin |
| evm.model.scaffold00007.30 | 1.11.1.9 | glutathione peroxidase |
| evm.model.scaffold00511.1 | 1.14.13.11 | trans-cinnamate 4-monooxygenase |
| evm.model.scaffold00013.43 | 1.14.14.1 | unspecific monooxygenase |
| evm.model.scaffold00122.18 | 2.3.1.48 | histone acetyltransferase |
| evm.model.scaffold00122.19 | 2.3.1.48 | histone acetyltransferase |
| evm.model.scaffold00075.16 | 2.3.1.79 | maltose O-acetyltransferase |
| evm.model.scaffold00117.22 | 2.4.1.17 | glucuronosyltransferase |
| evm.model.scaffold00398.2 | 2.4.2.29 | tRNA-guanosine34 transglycosylase |
| evm.model.scaffold00212.16 | 2.5.1.18 | glutathione transferase |
| evm.model.scaffold00006.78 | 2.7.1.150 | 1-phosphatidylinositol-3-phosphate 5-kinase |
| evm.model.scaffold00206.1 | 2.7.1.33 | pantothenate kinase |
| evm.model.scaffold00592.1 | 2.7.1.33 | pantothenate kinase |
| evm.model.scaffold00392.2 | 2.7.1.48 | uridine kinase |
| evm.model.scaffold00009.81 | 2.7.11.1 | non-specific serine/threonine protein kinase |
| evm.model.scaffold00038.13 | 2.7.11.1 | non-specific serine/threonine protein kinase |
| evm.model.scaffold00038.47 | 2.7.11.1 | non-specific serine/threonine protein kinase |
| evm.model.scaffold00041.35 | 2.7.11.1 | non-specific serine/threonine protein kinase |
| evm.model.scaffold00042.27 | 2.7.11.1 | non-specific serine/threonine protein kinase |
| evm.model.scaffold00093.10 | 2.7.11.1 | non-specific serine/threonine protein kinase |
| evm.model.scaffold00122.10 | 2.7.11.1 | non-specific serine/threonine protein kinase |
| evm.model.scaffold00217.19 | 2.7.11.1 | non-specific serine/threonine protein kinase |
| evm.model.scaffold00235.8 | 2.7.11.1 | non-specific serine/threonine protein kinase |
| evm.model.scaffold00317.3 | 2.7.11.1 | non-specific serine/threonine protein kinase |
| evm.model.scaffold00353.1 | 2.7.11.1 | non-specific serine/threonine protein kinase |
| evm.model.scaffold00529.2 | 2.7.11.1 | non-specific serine/threonine protein kinase |
| evm.model.scaffold00762.1 | 2.7.11.1 | non-specific serine/threonine protein kinase |
| evm.model.scaffold00039.47 | 2.7.11.13 | protein kinase C |
| evm.model.scaffold00214.16 | 2.7.11.13 | protein kinase C |
| evm.model.scaffold00214.18 | 2.7.11.13 | protein kinase C |
| evm.model.scaffold00014.16 | 2.7.11.17 | Ca^2+^/calmodulin-dependent protein kinase |
| evm.model.scaffold00467.2 | 2.7.11.2 | [pyruvate dehydrogenase (acetyl-transferring)] kinase |
| evm.model.scaffold00360.1 | 2.7.11.21 | polo kinase |
| evm.model.scaffold00231.7 | 2.7.11.22 | cyclin-dependent kinase |
| evm.model.scaffold00122.26 | 2.7.12.1 | dual-specificity kinase |
| evm.model.scaffold00040.28 | 2.7.13.3 | histidine kinase |
| evm.model.scaffold00040.30 | 2.7.13.3 | histidine kinase |
| evm.model.scaffold00063.30 | 2.7.4.3 | adenylate kinase |
| evm.model.scaffold00196.8 | 2.7.7.1 | nicotinamide-nucleotide adenylyltransferase |
| evm.model.scaffold00047.21 | 2.7.7.14 | ethanolamine-phosphate cytidylyltransferase |
| evm.model.scaffold00040.35 | 2.7.7.49 | RNA-directed DNA polymerase |
| evm.model.scaffold00078.1 | 2.7.7.50 | mRNA guanylyltransferase |
| evm.model.scaffold00101.24 | 2.7.7.6 | DNA-directed RNA polymerase |
| evm.model.scaffold00006.16 | 3.1.2.15 | ubiquitinyl hydrolase 1 |
| evm.model.scaffold00126.28 | 3.1.2.15 | ubiquitinyl hydrolase 1 |
| evm.model.scaffold00472.1 | 3.1.2.15 | ubiquitinyl hydrolase 1 |
| evm.model.scaffold00317.11 | 3.1.2.4 | 3-hydroxyisobutyryl-CoA hydrolase |
| evm.model.scaffold00141.28 | 3.1.2.6 | hydroxyacylglutathione hydrolase |
| evm.model.scaffold00048.32 | 3.1.26.4 | ribonuclease H |
| evm.model.scaffold00146.6 | 3.1.3.11 | fructose-1,6-bisphosphatase |
| evm.model.scaffold00118.35 | 3.1.4.35 | 3',5'-cyclic-GMP phosphodiesterase |
| evm.model.scaffold00127.32 | 3.2.1.113 | mannosyl-oligosaccharide 1,2-alpha-mannosidase |
| evm.model.scaffold00103.28 | 3.2.1.51 | alpha-L-fucosidase |
| evm.model.scaffold00001.79 | 3.3.2.1 | isochorismatase |
| evm.model.scaffold00806.1 | 3.4.17.21 | glutamate carboxypeptidase II |
| evm.model.scaffold00216.19 | 3.4.19.12 | ubiquitinyl hydrolase 1 |
| evm.model.scaffold00031.59 | 3.4.21.48 | cerevisin |
| evm.model.scaffold00148.32 | 3.4.21.83 | oligopeptidase B |
| evm.model.scaffold00120.23 | 3.4.22.49 | separase |
| evm.model.scaffold00185.22 | 3.5.1.98 | histone deacetylase |
| evm.model.scaffold00383.5 | 3.6.1.1 | inorganic diphosphatase |
| evm.model.scaffold00188.3 | 3.6.1.10 | endopolyphosphatase |
| evm.model.scaffold00318.4 | 3.6.3.14 | H^+^-transporting two-sector ATPase |
| evm.model.scaffold00633.1 | 3.6.3.14 | H^+^-transporting two-sector ATPase |
| evm.model.scaffold00017.13 | 3.6.3.6 | H^+^-exporting ATPase |
| evm.model.scaffold00017.14 | 3.6.3.6 | H^+^-exporting ATPase |
| evm.model.scaffold00206.5 | 3.6.3.6 | H^+^-exporting ATPase |
| evm.model.scaffold00315.5 | 3.6.3.6 | H^+^-exporting ATPase |
| evm.model.scaffold00082.16 | 3.6.4.12 | DNA helicase |
| evm.model.scaffold00173.1 | 3.6.4.12 | DNA helicase |
| evm.model.scaffold00019.45 | 3.6.4.13 | RNA helicase |
| evm.model.scaffold00045.13 | 3.6.4.13 | RNA helicase |
| evm.model.scaffold00156.18 | 3.6.4.13 | RNA helicase |
| evm.model.scaffold00241.15 | 3.6.4.13 | RNA helicase |
| evm.model.scaffold00353.3 | 3.6.4.13 | RNA helicase |
| evm.model.scaffold00548.2 | 3.6.4.13 | RNA helicase |
| evm.model.scaffold00681.2 | 3.6.5.5 | dynamin GTPase |
| evm.model.scaffold00011.18 | 3.8.1.2 | (S)-2-haloacid dehalogenase |
| evm.model.scaffold00036.54 | 4.1.1.21 | phosphoribosylaminoimidazole carboxylase |
| evm.model.scaffold00213.12 | 4.1.1.35 | UDP-glucuronate decarboxylase |
| evm.model.scaffold00167.6 | 4.1.1.74 | indolepyruvate decarboxylase |
| evm.model.scaffold00015.6 | 4.3.1.17 | L-serine ammonia-lyase |
| evm.model.scaffold00320.5 | 5.2.1.8 | peptidylprolyl isomerase |
| evm.model.scaffold00109.10 | 6.1.1.7 | alanine-tRNA ligase |
| evm.model.scaffold00028.15 | 6.3.1.2 | glutamate-ammonia ligase |
| evm.model.scaffold00001.11 | 6.3.2.19 | ubiquitin-protein ligase |
| evm.model.scaffold00009.13 | 6.3.2.19 | ubiquitin-protein ligase |
| evm.model.scaffold00009.14 | 6.3.2.19 | ubiquitin-protein ligase |
| evm.model.scaffold00021.67 | 6.3.2.19 | ubiquitin-protein ligase |
| evm.model.scaffold00024.4 | 6.3.2.19 | ubiquitin-protein ligase |
| evm.model.scaffold00092.14 | 6.3.2.19 | ubiquitin-protein ligase |
| evm.model.scaffold00126.13 | 6.3.2.19 | ubiquitin-protein ligase |
| evm.model.scaffold00126.31 | 6.3.2.19 | ubiquitin-protein ligase |
| evm.model.scaffold00160.6 | 6.3.2.19 | ubiquitin-protein ligase |
| evm.model.scaffold00193.2 | 6.3.2.19 | ubiquitin-protein ligase |
| evm.model.scaffold00202.13 | 6.3.2.19 | ubiquitin-protein ligase |
| evm.model.scaffold00110.7 | 6.3.5.5 | carbamoyl-phosphate synthase (glutamine-hydrolysing) |
