# Supplementary material for: Complete Genome Sequence of a High Lipid-Producing Strain of Mucor circinelloides WJ11 and Comparative Genome Analysis with a Low Lipid-Producing Strain CBS 277.49
Source: PLoS One. 2015 Sep 9;10(9):e0137543. doi: 10.1371/journal.pone.0137543 (PMC4564205; doi:10.1371/journal.pone.0137543)
Supplement: S4 Table — (DOCX) [file pone.0137543.s004.docx]

**S4 Table. Listing of *M. circinelloides* CBS 277.49 specific genes as compared to *M. circinelloides* WJ11.**

| **Gene ID** | **EC number** | **Enzyme** |
| --- | --- | --- |
| **Cell growth related genes** |  |  |
| jgi\|Mucci2\|154996\| | 2.4.1.16 | chitin synthase |
| jgi\|Mucci2\|154997\| | 2.4.1.16 | chitin synthase |
| jgi\|Mucci2\|112521\| | 3.6.3.8 | Ca^2+^-transporting ATPase |
| **Carbohydrate metabolism** |  |  |
| jgi\|Mucci2\|157406\| | 1.1.1.169 | 2-dehydropantoate 2-reductase |
| jgi\|Mucci2\|157407\| | 1.1.1.169 | 2-dehydropantoate 2-reductase |
| jgi\|Mucci2\|79453\| | 1.2.4.1 | pyruvate dehydrogenase |
| jgi\|Mucci2\|155844\| | 1.2.1.12 | glyceraldehyde-3-phosphate dehydrogenase |
| jgi\|Mucci2\|155816\| | 1.3.5.1 | succinate dehydrogenase |
| jgi\|Mucci2\|32094\| | 1.3.5.1 | succinate dehydrogenase |
| jgi\|Mucci2\|157169\| | 2.3.1.12 | dihydrolipoyllysine acetyltransferase |
| jgi\|Mucci2\|157170\| | 2.3.1.12 | dihydrolipoyllysine acetyltransferase |
| jgi\|Mucci2\|156948\| | 2.3.1.61 | dihydrolipoyllysine succinyltransferase |
| jgi\|Mucci2\|173013\| | 2.4.1.1 | glycogen phosphorylase |
| jgi\|Mucci2\|152756\| | 2.4.1.1 | glycogen phosphorylase |
| jgi\|Mucci2\|156043\| | 2.4.1.17 | glucuronosyltransferase |
| jgi\|Mucci2\|164856\| | 2.7.1.17 | xylulokinase |
| jgi\|Mucci2\|156910\| | 2.7.1.40 | pyruvate kinase |
| jgi\|Mucci2\|73446\| | 2.7.2.3 | phosphoglycerate kinase |
| jgi\|Mucci2\|139204\| | 2.7.7.12 | UDP-glucose-hexose-1-phosphate uridylyltransferase |
| jgi\|Mucci2\|155835\| | 2.7.7.13 | mannose-1-phosphate guanylyltransferase |
| jgi\|Mucci2\|161275\| | 3.5.1.25 | N-acetylglucosamine-6-phosphate deacetylase |
| jgi\|Mucci2\|154750\| | 6.2.1.4 | succinyl-CoA ligase |
| **Lipid metabolism** |  |  |
| jgi\|Mucci2\|157207\| | 1.1.1.8 | glycerol-3-phosphate dehydrogenase (NAD^+^) |
| jgi\|Mucci2\|157548\| | 1.14.14.1 | unspecific monooxygenase |
| jgi\|Mucci2\|179406\| | 1.14.14.1 | unspecific monooxygenase |
| jgi\|Mucci2\|157229\| | 1.3.1.70 | Delta14-sterol reductase |
| jgi\|Mucci2\|113163\| | 1.3.1.71 | Delta24(241)-sterol reductase |
| jgi\|Mucci2\|131688\| | 1.3.99.5 | 3-oxo-5alpha-steroid 4-dehydrogenase (acceptor) |
| jgi\|Mucci2\|155675\| | 1.3.99.5 | 3-oxo-5alpha-steroid 4-dehydrogenase |
| jgi\|Mucci2\|108531\| | 2.3.1.15 | glycerol-3-phosphate 1-O-acyltransferase |
| jgi\|Mucci2\|168275\| | 2.3.1.16 | 3-ketoacyl-CoA thiolase |
| jgi\|Mucci2\|156700\| | 2.3.1.51 | 1-acylglycerol-3-phosphate O-acyltransferase |
| jgi\|Mucci2\|155655\| | 2.7.7.14 | ethanolamine-phosphate cytidylyltransferase |
| jgi\|Mucci2\|175519\| | 2.7.7.15 | choline-phosphate cytidylyltransferase |
| jgi\|Mucci2\|82376\| | 2.7.7.41 | phosphatidate cytidylyltransferase |
| jgi\|Mucci2\|140608\| | 3.1.1.3 | triacylglycerol lipase |
| **Others** |  |  |
| jgi\|Mucci2\|156333\| | 1.1.1.95 | phosphoglycerate dehydrogenase |
| jgi\|Mucci2\|155577\| | 1.1.99.1 | choline dehydrogenase |
| jgi\|Mucci2\|137247\| | 1.6.2.2 | cytochrome-b5 reductase |
| jgi\|Mucci2\|155973\| | 1.9.3.1 | cytochrome-c oxidase |
| jgi\|Mucci2\|19138\| | 1.10.3.2 | laccase |
| jgi\|Mucci2\|125882\| | 1.16.3.1 | ferroxidase |
| jgi\|Mucci2\|115187\| | 1.20.4.1 | arsenate reductase (glutaredoxin) |
| jgi\|Mucci2\|158919\| | 1.5.1.15 | methylenetetrahydrofolate dehydrogenase (NAD+) |
| jgi\|Mucci2\|121364\| | 1.6.2.4 | NADPH-hemoprotein reductase |
| jgi\|Mucci2\|122685\| | 1.6.2.4 | NADPH-hemoprotein reductase |
| jgi\|Mucci2\|147670\| | 1.6.2.4 | NADPH-hemoprotein reductase |
| jgi\|Mucci2\|151217\| | 1.6.5.3 | NADH:ubiquinone reductase (H^+^-translocating) |
| jgi\|Mucci2\|151220\| | 1.6.5.3 | NADH:ubiquinone reductase (H^+^-translocating) |
| jgi\|Mucci2\|151225\| | 1.6.5.3 | NADH:ubiquinone reductase (H^+^-translocating) |
| jgi\|Mucci2\|151229\| | 1.6.5.3 | NADH:ubiquinone reductase (H^+^-translocating) |
| jgi\|Mucci2\|155490\| | 1.8.1.7 | glutathione-disulfide reductase |
| jgi\|Mucci2\|149430\| | 1.8.1.9 | thioredoxin-disulfide reductase |
| jgi\|Mucci2\|86847\| | 2.1.1.37 | DNA (cytosine-5-)-methyltransferase |
| jgi\|Mucci2\|151156\| | 2.1.1.43 | histone-lysine N-methyltransferase |
| jgi\|Mucci2\|34233\| | 2.1.1.43 | histone-lysine N-methyltransferase |
| jgi\|Mucci2\|156675\| | 2.1.1.77 | protein-L-isoaspartate(D-aspartate) O-methyltransferase |
| jgi\|Mucci2\|105145\| | 2.3.1.128 | ribosomal-protein-alanine N-acetyltransferase |
| jgi\|Mucci2\|154800\| | 2.3.1.22 | 2-acylglycerol O-acyltransferase |
| jgi\|Mucci2\|154801\| | 2.3.1.22 | 2-acylglycerol O-acyltransferase |
| jgi\|Mucci2\|145948\| | 2.3.1.4 | glucosamine-phosphate N-acetyltransferase |
| jgi\|Mucci2\|141423\| | 2.3.1.48 | histone acetyltransferase |
| jgi\|Mucci2\|152172\| | 2.3.1.48 | histone acetyltransferase |
| jgi\|Mucci2\|48472\| | 2.3.1.48 | histone acetyltransferase |
| jgi\|Mucci2\|75234\| | 2.3.1.48 | histone acetyltransferase |
| jgi\|Mucci2\|39631\| | 2.4.1.141 | N-acetylglucosaminyldiphosphodolichol N-acetylglucosaminyltransferase |
| jgi\|Mucci2\|49883\| | 2.4.1.141 | N-acetylglucosaminyldiphosphodolichol N-acetylglucosaminyltransferase |
| jgi\|Mucci2\|156043\| | 2.4.1.17 | glucuronosyltransferase |
| jgi\|Mucci2\|125379\| | 2.4.2.30 | NAD^+^ ADP-ribosyltransferase |
| jgi\|Mucci2\|157599\| | 2.5.1.16 | spermidine synthase |
| jgi\|Mucci2\|141306\| | 2.5.1.17 | cob(I)yrinic acid a,c-diamide adenosyltransferase |
| jgi\|Mucci2\|163042\| | 2.5.1.18 | glutathione transferase |
| jgi\|Mucci2\|72305\| | 2.5.1.18 | glutathione transferase |
| jgi\|Mucci2\|19365\| | 2.7.1.150 | 1-phosphatidylinositol-3-phosphate 5-kinase |
| jgi\|Mucci2\|179951\| | 2.7.1.26 | riboflavin kinase |
| jgi\|Mucci2\|157349\| | 2.7.1.33 | pantothenate kinase |
| jgi\|Mucci2\|109224\| | 2.7.11.1 | non-specific serine/threonine protein kinase |
| jgi\|Mucci2\|113411\| | 2.7.11.1 | non-specific serine/threonine protein kinase |
| jgi\|Mucci2\|125413\| | 2.7.11.1 | non-specific serine/threonine protein kinase |
| jgi\|Mucci2\|125432\| | 2.7.11.1 | non-specific serine/threonine protein kinase |
| jgi\|Mucci2\|137370\| | 2.7.11.1 | non-specific serine/threonine protein kinase |
| jgi\|Mucci2\|140977\| | 2.7.11.1 | non-specific serine/threonine protein kinase |
| jgi\|Mucci2\|144611\| | 2.7.11.1 | non-specific serine/threonine protein kinase |
| jgi\|Mucci2\|146462\| | 2.7.11.1 | non-specific serine/threonine protein kinase |
| jgi\|Mucci2\|147921\| | 2.7.11.1 | non-specific serine/threonine protein kinase |
| jgi\|Mucci2\|149405\| | 2.7.11.1 | non-specific serine/threonine protein kinase |
| jgi\|Mucci2\|155028\| | 2.7.11.1 | non-specific serine/threonine protein kinase |
| jgi\|Mucci2\|155258\| | 2.7.11.1 | non-specific serine/threonine protein kinase |
| jgi\|Mucci2\|156022\| | 2.7.11.1 | non-specific serine/threonine protein kinase |
| jgi\|Mucci2\|156730\| | 2.7.11.1 | non-specific serine/threonine protein kinase |
| jgi\|Mucci2\|157358\| | 2.7.11.1 | non-specific serine/threonine protein kinase |
| jgi\|Mucci2\|157861\| | 2.7.11.1 | non-specific serine/threonine protein kinase |
| jgi\|Mucci2\|163567\| | 2.7.11.1 | non-specific serine/threonine protein kinase |
| jgi\|Mucci2\|164694\| | 2.7.11.1 | non-specific serine/threonine protein kinase |
| jgi\|Mucci2\|166865\| | 2.7.11.1 | non-specific serine/threonine protein kinase |
| jgi\|Mucci2\|42753\| | 2.7.11.1 | non-specific serine/threonine protein kinase |
| jgi\|Mucci2\|150514\| | 2.7.4.3 | adenylate kinase |
| jgi\|Mucci2\|147857\| | 2.7.4.6 | nucleoside-diphosphate kinase |
| jgi\|Mucci2\|110210\| | 2.7.6.2 | thiamine diphosphokinase |
| jgi\|Mucci2\|110626\| | 2.7.7.1 | nicotinamide-nucleotide adenylyltransferase |
| jgi\|Mucci2\|149592\| | 2.7.7.6 | DNA-directed RNA polymerase |
| jgi\|Mucci2\|45209\| | 2.7.7.6 | DNA-directed RNA polymerase |
| jgi\|Mucci2\|154342\| | 2.7.8.1 | ethanolaminephosphotransferase |
| jgi\|Mucci2\|114165\| | 2.7.8.7 | holo-[acyl-carrier-protein] synthase |
| jgi\|Mucci2\|148087\| | 3.1.1.1 | carboxylesterase |
| jgi\|Mucci2\|114537\| | 3.1.1.29 | aminoacyl-tRNA hydrolase |
| jgi\|Mucci2\|114070\| | 3.1.2.15 | ubiquitinyl hydrolase 1 |
| jgi\|Mucci2\|118206\| | 3.1.2.15 | ubiquitinyl hydrolase 1 |
| jgi\|Mucci2\|133428\| | 3.1.2.15 | ubiquitinyl hydrolase 1 |
| jgi\|Mucci2\|14515\| | 3.1.2.15 | ubiquitinyl hydrolase 1 |
| jgi\|Mucci2\|161784\| | 3.1.2.4 | 3-hydroxyisobutyryl-CoA hydrolase |
| jgi\|Mucci2\|36529\| | 3.1.2.4 | 3-hydroxyisobutyryl-CoA hydrolase |
| jgi\|Mucci2\|157950\| | 3.1.26.4 | ribonuclease H |
| jgi\|Mucci2\|110658\| | 3.1.26.5 | ribonuclease P |
| jgi\|Mucci2\|114779\| | 3.1.26.5 | ribonuclease P |
| jgi\|Mucci2\|116093\| | 3.1.26.5 | ribonuclease P |
| jgi\|Mucci2\|164345\| | 3.1.27.9 | tRNA-intron lyase |
| jgi\|Mucci2\|154371\| | 3.1.3.16 | protein-serine/threonine phosphatase |
| jgi\|Mucci2\|157329\| | 3.1.3.16 | protein-serine/threonine phosphatase |
| jgi\|Mucci2\|184375\| | 3.1.3.16 | protein-serine/threonine phosphatase |
| jgi\|Mucci2\|82539\| | 3.1.3.16 | protein-serine/threonine phosphatase |
| jgi\|Mucci2\|108538\| | 3.1.3.3 | phosphoserine phosphatase |
| jgi\|Mucci2\|119955\| | 3.1.3.48 | protein-tyrosine-phosphatase |
| jgi\|Mucci2\|136959\| | 3.1.3.48 | protein-tyrosine-phosphatase |
| jgi\|Mucci2\|170535\| | 3.1.3.48 | protein-tyrosine-phosphatase |
| jgi\|Mucci2\|112349\| | 3.1.3.67 | phosphatidylinositol-3,4,5-trisphosphate 3-phosphatase |
| jgi\|Mucci2\|140733\| | 3.1.4.17 | 3',5'-cyclic-nucleotide phosphodiesterase |
| jgi\|Mucci2\|148944\| | 3.2.1.51 | alpha-L-fucosidase |
| jgi\|Mucci2\|166501\| | 3.3.2.1 | isochorismatase |
| jgi\|Mucci2\|181540\| | 3.4.13.9 | Xaa-Pro dipeptidase |
| jgi\|Mucci2\|114194\| | 3.4.19.12 | ubiquitinyl hydrolase 1 |
| jgi\|Mucci2\|120703\| | 3.4.21.48 | cerevisin |
| jgi\|Mucci2\|156405\| | 3.4.21.48 | cerevisin |
| jgi\|Mucci2\|93158\| | 3.4.23.24 | candidapepsin |
| jgi\|Mucci2\|155375\| | 3.4.23.25 | saccharopepsin |
| jgi\|Mucci2\|153075\| | 3.4.23.5 | cathepsin D |
| jgi\|Mucci2\|37597\| | 3.5.1.52 | peptide-N4-(N-acetyl-beta-glucosaminyl) asparagine amidase |
| jgi\|Mucci2\|154688\| | 3.5.1.98 | histone deacetylase |
| jgi\|Mucci2\|155588\| | 3.5.3.1 | arginase |
| jgi\|Mucci2\|106235\| | 3.5.4.3 | guanine deaminase |
| jgi\|Mucci2\|116165\| | 3.6.1.29 | bis(5'-adenosyl)-triphosphatase |
| jgi\|Mucci2\|85798\| | 3.6.1.29 | bis(5'-adenosyl)-triphosphatase |
| jgi\|Mucci2\|154648\| | 3.6.3.14 | H+-transporting two-sector ATPase |
| jgi\|Mucci2\|156121\| | 3.6.3.14 | H+-transporting two-sector ATPase |
| jgi\|Mucci2\|113380\| | 3.6.4.12 | DNA helicase |
| jgi\|Mucci2\|115288\| | 3.6.4.12 | DNA helicase |
| jgi\|Mucci2\|158561\| | 3.6.4.12 | DNA helicase |
| jgi\|Mucci2\|78281\| | 3.6.4.12 | DNA helicase |
| jgi\|Mucci2\|121778\| | 3.6.4.13 | RNA helicase |
| jgi\|Mucci2\|128375\| | 3.6.4.13 | RNA helicase |
| jgi\|Mucci2\|153487\| | 3.6.4.13 | RNA helicase |
| jgi\|Mucci2\|156151\| | 3.6.4.13 | RNA helicase |
| jgi\|Mucci2\|156434\| | 3.6.4.13 | RNA helicase |
| jgi\|Mucci2\|156650\| | 3.6.4.13 | RNA helicase |
| jgi\|Mucci2\|117953\| | 3.6.5.5 | dynamin GTPase |
| jgi\|Mucci2\|109115\| | 4.1.1.36 | phosphopantothenoylcysteine decarboxylase |
| jgi\|Mucci2\|144082\| | 4.1.1.46 | o-pyrocatechuate decarboxylase |
| jgi\|Mucci2\|153742\| | 4.1.1.46 | o-pyrocatechuate decarboxylase |
| jgi\|Mucci2\|110193\| | 4.1.1.48 | indole-3-glycerol-phosphate synthase |
| jgi\|Mucci2\|150848\| | 4.2.1.104 | cyanase |
| jgi\|Mucci2\|139911\| | 4.3.1.17 | L-serine ammonia-lyase |
| jgi\|Mucci2\|154457\| | 4.3.2.1 | argininosuccinate lyase |
| jgi\|Mucci2\|114427\| | 5.2.1.8 | peptidylprolyl isomerase |
| jgi\|Mucci2\|156077\| | 5.2.1.8 | peptidylprolyl isomerase |
| jgi\|Mucci2\|154978\| | 5.2.1.8 | peptidylprolyl isomerase |
| jgi\|Mucci2\|109216\| | 5.3.1.23 | S-methyl-5-thioribose-1-phosphate isomerase |
| jgi\|Mucci2\|35246\| | 5.3.4.1 | protein disulfide-isomerase |
| jgi\|Mucci2\|156356\| | 6.1.1.20 | phenylalanine-tRNA ligase |
| jgi\|Mucci2\|105829\| | 6.1.1.7 | alanine-tRNA ligase |
| jgi\|Mucci2\|154694\| | 6.3.1.2 | glutamate-ammonia ligase |
| jgi\|Mucci2\|110918\| | 6.3.2.19 | ubiquitin-protein ligase |
| jgi\|Mucci2\|120214\| | 6.3.2.19 | ubiquitin-protein ligase |
| jgi\|Mucci2\|124118\| | 6.3.2.19 | ubiquitin-protein ligase |
| jgi\|Mucci2\|137701\| | 6.3.2.19 | ubiquitin-protein ligase |
| jgi\|Mucci2\|144472\| | 6.3.2.19 | ubiquitin-protein ligase |
| jgi\|Mucci2\|148078\| | 6.3.2.19 | ubiquitin-protein ligase |
| jgi\|Mucci2\|155132\| | 6.3.2.19 | ubiquitin-protein ligase |
| jgi\|Mucci2\|156820\| | 6.3.2.19 | ubiquitin-protein ligase |
| jgi\|Mucci2\|162084\| | 6.3.2.19 | ubiquitin-protein ligase |
| jgi\|Mucci2\|165339\| | 6.3.2.19 | ubiquitin-protein ligase |
| jgi\|Mucci2\|24324\| | 6.3.2.19 | ubiquitin-protein ligase |
| jgi\|Mucci2\|30166\| | 6.3.2.19 | ubiquitin-protein ligase |
| jgi\|Mucci2\|74915\| | 6.3.2.19 | ubiquitin-protein ligase |
| jgi\|Mucci2\|81456\| | 6.3.2.19 | ubiquitin-protein ligase |
| jgi\|Mucci2\|91767\| | 6.3.2.19 | ubiquitin-protein ligase |
| jgi\|Mucci2\|155828\| | 6.3.5.5 | carbamoyl-phosphate synthase (glutamine-hydrolysing) |
